# Postoperative Pain Assessment Indices Based on Photoplethysmography Waveform Analysis

**DOI:** 10.3389/fphys.2018.01199

**Published:** 2018-08-28

**Authors:** Yoon La Yang, Hyeon Seok Seok, Gyu-Jeong Noh, Byung-Moon Choi, Hangsik Shin

**Affiliations:** ^1^Interdisciplinary Program in Biomedical Engineering, Chonnam National University, Gwangju, South Korea; ^2^Department of Biomedical Engineering, Chonnam National University, Yeosu, South Korea; ^3^Department of Clinical Pharmacology and Therapeutics, University of Ulsan College of Medicine, Seoul, South Korea; ^4^Department of Anesthesiology and Pain Medicine, Asan Medical Center, University of Ulsan College of Medicine, Seoul, South Korea

**Keywords:** anesthesia, pain quantification, photoplethysmography, post-operative pain, surgical pain

## Abstract

The purpose of this study was to derive parameters that might reflect postoperative pain from photoplethysmography (PPG) and verify the derived parameters in postoperative pain assessment. We obtained preoperative and postoperative PPG and 100-mm visual analog scale (VAS) from 65 surgical patients and extracted a total of 51 PPG morphology-based parameters and their normalized parameters from these PPGs obtained. Pain discrimination performances of these derived parameters were assessed by statistical analyses, including Wilcoxon signed rank test with Bonferroni correction, classification accuracy based on logistic regression, and 4-fold cross validation. After comparing these parameters derived from PPG in pre- and post-operative conditions, statistically significant difference was found in 36 of the 51 parameters. Using logistic classification, dynamic between-pulse parameters such as normalized systolic amplitude variation and normalized diastolic amplitude variation showed better pain classification performance than the static within-pulse parameters. VAS score was 0 in every pre-operation condition, but >60 VAS was observed in the post-operative condition. Systolic peak amplitude variation normalized by PPG AC amplitude showed the best performance in classifying post-operative pain, with accuracy, sensitivity, specificity, and positive predictivity values of 79.5, 74.0, 86.0, and 84.5%, respectively. These results are superior to those of the surgical pleth index (SPI, GE Healthcare, Chicago, IL, United States) at 65.9, 65.9, 66.5, and 66.5%, respectively.

## Introduction

Up to 50% of surgical patients are known to experience moderate or even severe pain immediately after surgery (Boselli et al., 2013; Gerbershagen et al., 2013). While opioids are widely used as effective analgesics, opioid sensitivity is well known to vary widely among individuals. Pain sensitivity and analgesic susceptibility are major factors limiting the objectivity of post-operative pain management. Opioids can result in underdose or overdose of medication. Overdosing of analgesics is a life-threatening condition with disadvantageous effects such as prolonged sedation or prolonged respiratory insufficiency (Macintyre et al., 2011; Apfel et al., 2012). In addition, opioid analgesic overdose encompasses a range of clinical findings such as respiratory depression, miosis, stupor, hepatic injury from acetaminophen or hypoxemia, myoglobinuric renal failure, rhabdomyolysis, absent or hypoactive bowel sounds, compartment syndrome, and hypothermia (Boyer, 2012).

Therefore, the challenge for an effective analgesic strategy is to estimate the dose that can exactly counterbalance the amount of ongoing nociception with respect to individual susceptibility to analgesics and individual sensitivity to pain (Ip et al., 2009). Maintaining an appropriate level of analgesics is very important to improve the patient’s prognosis (Musizza and Ribaric, 2010). However, it is difficult to judge the adequate amount of opioid because the degree of pain experienced during surgery varies depending on the patient as well as the environmental and emotional factors of the same patient. This is because indices such as anxiety, sensitivity, and distress tolerance closely related to pain experience can vary greatly among individuals.

To quantify surgical pain, previous studies have focused on autonomic nervous system responses such as changes in heart rate (HR) and blood pressure, amount of tears, changes in pupil size, respiratory rate and volume, and patient movement (Russell, 1989; Moerman et al., 1993). Among these responses, the surgical pleth index (SPI, GE Healthcare, Chicago, IL, United States), an index based on photoplethysmography (PPG), is a promising surgical pain quantification index. SPI is a non-invasive, dimensionless score (0–100), which allows an estimate of intraoperative nociception. SPI is a model of PPG amplitude (PPGA) and heart beat interval (HBI). It is modeled as SPI = 100 × (0.67 × PPGA_norm_+0.33 × HBI_norm_) (Huiku et al., 2007). PPGA is known to be strongly correlated with sympathetic response by surgical stimulation while HBI is known to correlate with opioid effect (Huiku et al., 2007). The value of SPI ranges from 0 to 100, with a value of 0 indicating weaker surgical stimulus, while a value closer to 100 indicated stronger surgical stimulus. In addition, SPI 50 can be used to differentiate a patient’s pain intensity in general anesthesia (Chen et al., 2010). It has been reported that SPI can vary with pain-stimuli or anesthetic concentration in propofol-remifentanil anesthesia (Huiku et al., 2007). SPI has demonstrated excellent performance compared to various other pain estimation methods based on HR, blood pressure, or response entropy (Wennervirta et al., 2008). Several studies have reported that SPI is a potential indicator for pain assessment in unconscious patients (Ahonen et al., 2007; Huiku et al., 2007; Kallio et al., 2008). However, according to Ledowski et al. (2009), SPI has limited accuracy in assessing conscious subjects. In fact, the manufacturer recommends the use of SPI only during anesthesia; this is presumably because SPI is normalized with data from anesthesia patients only. At present, SPI are frequently used in the clinical setting. However, a complete assessment technique for pain is not available, especially for post-operative pain.

In this study, we proposed new indicators in post-operative pain assessment by deriving characteristic features not presented in previous studies through PPG waveform analysis. We then verified the pain correlation of the derived candidate parameters. In particular, in the previous studies, pain caused by specific stimuli was evaluated, such as incision during surgery (Ahonen et al., 2007; Huiku et al., 2007; Kallio et al., 2008; Boselli et al., 2013), but this may include changes due to various factors such as the effect of an electrosurgical unit other than the pain itself. Moreover, in a clinical setting, it is important to distinguish pain from non-pain because the specified dose is repeatedly administered depending on the presence or absence of pain without adjusting the dose according to the pain intensity. Therefore, in order to observe the pure changes due to pain, in this study, two conditions were defined rather than the various pain intensities: preoperative condition as a state without pain and post-operative condition as a state of pain.

## Materials and Methods

### Ethics and Dataset

This study was approved by the Asan Medical Center Institutional Review Board (approval number: 2016-0477) and registered on the international clinical trials registry platform (^1^KCT0002080). A total of 81 subjects were enrolled. All experiments were approved by the Human Research Ethics Committee of Asan Medical Center and all methods were performed in accordance with the relevant guidelines and regulations. All participants gave written informed consent for their participation in the study. Finally, data from 65 subjects (28 males, 37 females, mean age of 50.5 ± 10.5 years) were used for analysis, excluding data from 16 subjects: data from 2 subjects had recording errors, 1 subject had severe arrhythmia; and data from 13 subjects were recorded without SPI. Patient’s information and type of surgery are described in **Table 1**.

**Table 1:** Patient characteristics and type of surgery.

	Number of patient
Total	65 (age: 50.5 ± 10.5)
Gender	
Male	28 (age: 54.1 ± 9.1)
Female	37 (age: 47.8 ± 10.7)
Diagnosis	
Cancer	56
Others^∗^	9
Operation	
BR	15
CRS	17
ES	6
HBP	7
ST	20

*^∗^Anastomosis stricture (*N* = 1), cholecystitis (*N* = 4), colon perforation (*N* = 1), inguinal hernia (*N* = 1), polyarteritis nodosa (*N* = 1), rectal polyp (*N* = 1), BR: breast surgery including breast conserving operation (*N* = 12), modified radical mastectomy (*N* = 3), CRS: colorectal surgery including abdominoperitoneal resection (*N* = 2), anterior resection (*N* = 1), colostomy (*N* = 2), ileostomy (*N* = 1), low anterior resection (*N* = 8), transanal dilatation (*N* = 1), transanal excision (*N* = 2), ES: thyroid surgery (*N* = 2), total thyroidectomy (*N* = 4), HBP: hepatobiliary surgery including distal pancreatectomy (*N* = 1), laparoscopic cholecystectomy (*N* = 4), left lobectomy of liver (*N* = 1), subphrenic mass excision (*N* = 1), ST: stomach surgery including herniorrhaphy (*N* = 1), laparoscopic gastrectomy (*N* = 12), open gastrectomy (*N* = 7).*

### Experimental Protocol

Photoplethysmography data were recorded at pre-operation and post-operation. All surgeries included skin incision, and were performed under general anesthesia to reduce surgical pain. Pre-operative data were recorded in the surgery waiting room immediately before surgery. First, the 100 mm visual analog scale (VAS) was recorded and the PPG and SPI were simultaneously recorded for 6 min. Post-operative data were recorded after transfer of the patient to the recovery room immediately after the operation. At this time, PPG and SPI were recorded for 6 min after first recording VAS. Opioid was not used for 6 min immediately after surgery, and it was administrated after signal measurement was completed.

The VAS consists of a straight line with endpoints defining the extreme limits of the signal. For pain intensity, the scale is most commonly anchored by “no pain” (score of 0) and “pain as bad as it could be” or “worst imaginable pain” [score of 100 (100 mm scale)] (Haefeli and Elfering, 2006). PPG was recorded on the index finger of the opposite arm with intravenous therapy at a sampling frequency of 300 Hz for 6 min in each period. SPI was simultaneously recorded every 10 s. A GE Datex-Ohmeda S/5 Anesthesia Monitor, disposable PPG sensor, and a portable computer running the S/5 Collect software (GE Healthcare, Chicago, IL, United States) were used for monitoring and recording, respectively. Every recording is performed at room temperature.

### Preprocessing

The obtained PPG was filtered using a finite impulse response (FIR) bandpass filter at 0.5–10 Hz. Systolic and diastolic peak points were detected using the adaptive threshold method (Shin et al., 2009). The detected peak position was verified by experienced researchers and manually corrected for false detected or missed peaks. In the case of post-operative measurements, motion artifact may also occur, which can distort the data, even rendering it impossible to analyze. Therefore, the section where motion noise occurred was manually excluded from the analysis. Matlab 2016b (Mathworks, Inc., MA, United States) was used for all procedures of peak detection and signal analysis.

### PPG Parameters

#### Basic Parameters

**FIGURE 1:**
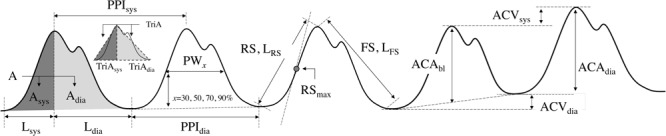
Graphical representation of extracted parameters from PPG morphology analysis.

Pain affects the autonomic nervous system, leading to the vasoconstriction of sympathetic nerves (Huiku et al., 2007). Most parameters of this study were derived based on this clinical fact. We derived 23 parameters from PPG morphology analysis. These parameters were based on the spatial characteristic, amplitude, temporal characteristic, and the length or interval of PPG. Each parameter for each condition was calculated by averaging the values obtained for each pulse for 6 min. Graphical representation of derived parameters is shown in **Figure 1**. Detailed descriptions are summarized in **Table 2**.

**Table 2:** Parameters extracted from PPG morphology analysis.

No.	Parameter name	Units	Definition	Description
1	A	a.u.	Area of a pulse	The area of a pulse
2	A_sys_	a.u.	Area of a systolic phase	The area of a pulse from the diastolic peak to the next systolic peak
3	A_dia_	a.u.	Area of a diastolic phase	The area of a pulse from the systolic peak to the next diastolic peak
4	TriA	a.u.	Triangular Area of a pulse	The area of the triangle made up of two adjacent diastole peaks and the systolic peak between them
5	TriA_sys_	a.u.	Triangular Area of a systolic phase	The area of triangle made up of diastolic peak, following systolic peak and intersection of the horizontal line of diastolic peak and vertical line of systolic peak
6	TriA_dia_	a.u.	Triangular Area of a diastolic phase	The area of triangle made up of systolic peak, following diastolic peak and intersection of the horizontal line of diastolic peak and vertical line of systolic peak
7	L_sys_	ms	Systolic length	The time interval between diastolic peak and next systolic peak
8	L_dia_	ms	Diastolic length	The time interval between systolic peak and next diastolic peak
9	PPI_sys_	ms	Systolic Peak Interval	The time interval between adjacent systolic peaks
10	PPI_dia_	ms	Diastolic Peak Interval	The time interval between adjacent diastolic peaks
11	ACA_dia_	a.u.	AC Amplitude from previous diastole	Difference of the systolic peak amplitude and the previous diastolic peak amplitude
12	ACA_bl_	a.u.	AC Amplitude from baseline	Difference of the systolic peak amplitude and the interpolated baseline amplitude of two adjacent diastolic peaks
13	ACV_sys_	a.u.	AC Variation Systole	Difference of the amplitude of systolic peaks
14	ACV_dia_	a.u.	AC Variation Diastole	Difference of the amplitude of diastolic peaks
15	RS	a.u.	Rising Slope	Slope between the diastolic peak and the next systolic peak
16	L_RS_	a.u.	Rising Slope Length	Distance between the diastolic peak and the next systolic peak
17	RS_max_	a.u.	Maximum Rising Slope	Maximum slope in the interval from the diastolic peak to the next systolic peak
18	FS	a.u.	Falling Slope	Slope between the systolic peak and the next diastolic peak
19	L_FS_	a.u.	Falling Slope Length	Distance between the systolic peak and the next diastolic peak
20–23	PW*_x_*	ms	Pulse Width at *x*% of maximum amplitude, *x* = 30, 50, 70, 90	Pulse width at *x*% point of maximum amplitude

#### Normalized Parameters

Amplitudes of PPG have arbitrary units (a.u.) because they are affected by various factors such as skin color of the subject, human body composition of the measurement site, anatomical structure, nail condition, ambient light, and sensor wearing condition at the time of measurement (Allen, 2007). Therefore, amplitude-based characteristics should not be quantified, and normalization for arbitrary units was considered. The parameters of the baseline-systolic amplitude of PPG (ACA_bl_) and the adjacent diastolic-systolic amplitude (ACA_dia_) had arbitrary units that required normalization. Area-related parameters related to ACA_bl_ and ACA_dia_ also had arbitrary units. Calculating the ratio between parameters including arbitrary units could give a dimensionless parameter instead of arbitrary units. Therefore, individual or measured conditional characteristics related to amplitude were removed. Time unit parameters are affected by HR, which has an inter-individual difference. Therefore, the inter-individual difference due to HR needs to be corrected. By calculating the ratio between time unit parameters, it is possible to eliminate inter-individual differences. Therefore, time unit parameters were normalized to adjacent diastolic peak interval or systolic peak-diastolic peak interval in the present study. Consequently, 28 normalized parameters including 27 non-dimensional parameters were derived by normalization. These normalized parameters are listed in **Table 3**.

**Table 3:** List of normalized parameters.

No.	Parameter name	Unit	Parameter definition
1	A_sys_/A	n.u.	Ratio of systolic area to pulse area
2	A_dia_/A	n.u.	Ratio of diastolic area to pulse area
3	A_sys_/A_dia_	n.u.	Ratio of systolic area to diastolic area
4	TriA_sys_/TriA	n.u.	Ratio of systolic triangle area to pulse triangle area
5	TriA_dia_/TriA	n.u.	Ratio of diastolic triangle area to pulse triangle area
6	TriA_sys_/TriA_dia_	n.u.	Ratio of systolic triangle area to diastolic triangle area
7	A/ACA_bl_	n.u.	Ratio of pulse area to AC amplitude
8	A_sys_/ACA_bl_	n.u.	Ratio of systolic area to AC amplitude
9	A_dia_/ACA_bl_	n.u.	Ratio of diastolic area to AC amplitude
10	L_sys_/PPI_f_	n.u.	Ratio of systolic length to foot peak interval
11	L_dia_/PPI_f_	n.u.	Ratio of diastolic length to foot peak interval
12	L_sys_/L_dia_	n.u.	Ratio of systolic length to diastolic length
13	ACV_sys_/ACA_dia_	n.u.	Ratio of AC variation systole to head peak height
14	ACV_sys_/ACA_bl_	n.u.	Ratio of AC variation systole to AC amplitude
15	ACV_dia_/ACA_dia_	n.u.	Ratio of AC variation diastole to head peak height
16	ACV_dia_/ACA_bl_	n.u.	Ratio of AC variation diastole to AC amplitude
17	RS_max_/RS	n.u.	Ratio of maximum rising slope to rising slope
18	RS/FS	n.u.	Ratio of rising slope to falling slope
19	RS/ACA_bl_	ms^-1^	Ratio of rising slope to AC amplitude
20	(A/ACA_bl_)/L_sys_	n.u.	Ratio of (A/ACA_bl_) to L_sys_
21	(A/ACA_bl_)/L_dia_	n.u.	Ratio of (A/ACA_bl_) to L_dia_
22	(A/ACA_bl_)/PPI_dia_	n.u.	Ratio of (A/ACA_bl_) to PPI_dia_
23	(A_sys_/ACA_bl_)/L_sys_	n.u.	Ratio of (A_sys_/ACA_bl_) to L_sys_
24	(A_sys_/ACA_bl_)/L_dia_	n.u.	Ratio of (A_sys_/ACA_bl_) to L_dia_
25	(A_sys_/ACA_bl_)/PPI_dia_	n.u.	Ratio of (A_sys_/ACA_bl_) to PPI_dia_
26	(A_dia_/ACA_bl_)/L_sys_	n.u.	Ratio of (A_dia_/ACA_bl_) to L_sys_
27	(A_dia_/ACA_bl_)/L_dia_	n.u.	Ratio of (A_dia_/ACA_bl_) to L_dia_
28	(A_dia_/ACA_bl_)/PPI_dia_	n.u.	Ratio of (A_dia_/ACA_bl_) to PPI_dia_

### Logistic Classification

Logistic classification has been used in binary classification problems. Logistic function is a monotonic, continuous function between 0 and 1. Mathematical expression of logistic function is represented in Eq. 1. Sigmoid function is a representative example of logistic function (*L* = 1, *k* = 1, *x*_0_ = 0). In logistic classification, output is a probability. In our study, preoperative data were set as class 0 while post-operative data were set as class 1, and we used a 0.5 as a classification criterion of pain or non-pain assessment. This means that we classified the result is a pain if *f*(*x*) > 0.5, and non-pain if *f*(*x*) < 0.5.

(1) $$\begin{array}{c} f(x)=\frac{1}{1+e^{-k(x-x_{0})}} \end{array}$$

### Validation

Validation was obtained using two methods: statistical significance test and accuracy evaluation of pain classification. Statistical tests were performed to determine if the derived parameters had significant changes due to pain. In statistical analysis, proposed parameters extracted from data obtained in pre- and post-operation were analyzed for statistical significance. To determine inter-subject variance, we calculated the coefficient of variance (CV). For testing normality, the Kolmogorov–Smirnov test was used. Because all data do not have normality, we used the Wilcoxon signed rank test to verify the significant difference between parameters derived with PPG measured on pre- and post-operation. In multiple comparisons, the Bonferroni correction was used. *P* < 0.05 was considered statistically significant.

The accuracy of pain classification was evaluated by applying logistic classification. For the random partitioning of samples into a training set and a test set, we used 4-fold cross validation. Classification performance was evaluated based on accuracy (AC), sensitivity (SE), specificity (SP), and positive predictivity value (PPV). AC indicates the proportion of correctly classified numbers in the total case. SE is the probability that a classification result will indicate pain among those with pain. SE is the fraction of those without pain who have a negative classification result. PPV is the probability of actual pain among those classified as pain.

## Results

### Statistical Validation

In our experiment, we confirmed that 0 VAS was observed in the pre-operation condition, while >60 VAS was observed in the post-operative condition. **Table 4** shows the mean, SD, CV, and *P*-value for each parameter before and after surgery. The results showed that SPI was significantly increased after the operation. Among the derived parameters, area-based indices such as A, A_sys_, A_dia_, TriA, TriA_sys_, and TriA_dia_ were significantly decreased in the post-operative condition. Amplitude-based indices, ACA_dia_ and ACA_bl_ were also significantly decreased in the post-operation. For temporal indices, L_sys_ was significantly increased in post-operation while L_dia_ was not significantly changed. Slope-based parameters, RS, L_RS_, RS_max_, FS, and L_FS_ were decreased significantly in post-operation. Pulse widths, PW_30_, PW_50_, and PW_70_ showed no significant change. However, PW_90_ were increased significantly in post-operation. Normalized area-based indices, A_sys_/A, A_dia_/A, A_sys_/A_dia_, TriA_sys_/TriA, TriA_diai_/TriA, and TriA_dia_/TriA, were found to have significant differences among the derived dimensionless parameters. The area of systolic phase was relatively increased, while the area of diastolic phase was decreased in post-operation. Similarly, A_sys_/ACA_bl_ showed a significant increase in the post-operation, whereas A_dia_/ACA_bl_ was not significantly changed. All of the RS/FS and RS/ACA_bl_ calculated as slope ratios were significantly decreased in the post-operative condition. However, the ratio of maximum rising slope to average rising slope, RS_max_/RS, was not significantly changed. This might be due to an increase in the systolic interval caused by postoperative pain, consistent with the results of parameters derived from area ratio. In addition, parameters (A/ACA_bl_)/L_sys_, (A/ACA_bl_)/L_dia_, (A_sys_/ACA_bl_)/L_sys_, and (A_sys_/ACA_bl_)/L_dia_ showed significant differences between pre- and post-operation. Results showed that a total of 15 parameters (L_dia_, PPI_sys_, PPI_dia_, ACV_sys_, ACV_dia_, PW_30_, PW_50_, PW_70_, A/ACA_bl_, A_dia_/ACA_bl_, RS_max_/RS, (A/ACA_bl_)/L_sys_, (A/ACA_bl_)/PPI_dia_, (A_dia_/ACA_bl_)/L_dia_, and (A_dia_/ACA_bl_)/PPI_dia_) were not significantly changed after the operation. For parameters representing dynamic inter-pulse variation, a significant increase was observed in the parameters of ACV_sys_/ACA_dia_, ACV_sys_/ACA_bl_, ACV_dia_/ACA_dia_, and ACV_dia_/ACA_bl_. This suggests that the fluctuation of amplitude is increased with pain.

**Table 4:** Statistical changes of derived parameters according to pain.

No.	Parameter	Mean (Standard Deviation)	Pre-operation Post-operation	Coefficient of Variation (%)	Pre-operation Post-operation	*P*-value
1	SPI	45.14 (16.35)	59.53 (13.83)	36.2	23.2	^∗∗∗^
2	A	441.2 (296.9)	230.4 (194.6)	67.3	84.5	^∗∗∗^
3	A_sys_	117.5 (72.9)	69.9 (58.7)	62.1	83.9	^∗^
4	A_dia_	323.7 (226.7)	160.5 (137.9)	70.0	85.9	^∗∗∗^
5	TriA	1.728 (1.184)	0.887 (0.793)	68.5	89.3	^∗∗∗^
6	TriA_sys_	0.388 (0.241)	0.226 (0.194)	62.3	85.6	^∗∗^
7	TriA_dia_	1.340 (0.952)	0.661 (0.607)	71.0	91.8	^∗∗∗^
8	L_sys_	0.204 (0.015)	0.214 (0.017)	7.5	8.0	^∗∗∗^
9	L_dia_	0.677 (0.136)	0.620 (0.151)	20.1	24.3	NS
10	PPI_sys_	0.881 (0.139)	0.833 (0.157)	15.7	18.9	NS
11	PPI_dia_	0.881 (0.139)	0.833 (0.158)	15.7	18.9	NS
12	ACA_dia_	3.828 (2.452)	2.130 (1.875)	64.1	88.0	^∗∗^
13	ACA_bl_	3.828 (2.452)	2.130 (1.875)	64.1	88.0	^∗∗^
14	ACV_sys_	0.083 (0.054)	0.081 (0.061)	65.9	75.7	NS
15	ACV_dia_	0.063 (0.042)	0.072 (0.059)	67.5	82.7	NS
16	RS	19.01 (12.63)	10.10 (9.21)	66.5	91.2	^∗∗∗^
17	L_RS_	3.837 (2.446)	2.149 (1.867)	63.8	86.9	^∗∗^
18	RS_max_	0.105 (0.069)	0.056 (0.051)	66.3	90.9	^∗∗^
19	FS	–5.718 (3.672)	–3.637 (3.417)	(64.2)	(94.0)	^∗^
20	L_FS_	3.919 (2.407)	’2.275 (1.8172)	61.4	79.9	^∗∗^
21	PW_30_	0.539 (0.123)	0.481 (0.114)	22.8	23.7	NS
22	PW_50_	0.270 (0.043)	0.273 (0.041)	16.1	14.9	NS
23	PW_70_	0.178 (0.024)	0.185 (0.024)	13.5	12.9	NS
24	PW_90_	0.094 (0.012)	0.099 (0.013)	13.1	12.8	^∗^
25	A_sys_/A	0.278 (0.044)	0.310 (0.045)	15.8	14.6	^∗∗∗^
26	A_dia_/A	0.722 (0.044)	0.690 (0.045)	6.1	6.6	^∗∗∗^
27	A_sys_/A_dia_	0.392 (0.091)	0.457 (0.097)	23.3	21.2	^∗∗∗^
28	TriA_sys_/TriA	0.237 (0.040)	0.264 (0.044)	16.8	16.5	^∗∗∗^
29	TriA_dia_/TriA	0.763 (0.040)	0.736 (0.044)	5.2	5.9	^∗∗∗^
30	TriA_sys_/TriA_dia_	0.315 (0.072)	0.365 (0.082)	22.7	22.6	^∗∗∗^
31	A/ACA_bl_	113.9 (18.4)	110.6 (20.5)	16.2	18.5	NS
32	A_sys_/ACA_bl_	30.97 (2.80)	33.34 (3.39)	9.0	10.2	^∗∗∗^
33	A_dia_/ACA_bl_	82.95 (17.34)	77.21 (18.67)	20.9	24.2	NS
34	L_sys_/PPI_dia_	0.237 (0.040)	0.264 (0.044)	16.8	16.5	^∗∗∗^
35	L_dia_/PPI_dia_	0.763 (0.040)	0.736 (0.044)	5.2	5.9	^∗∗∗^
36	L_sys_/L_dia_	0.315 (0.072)	0.365 (0.082)	22.7	22.6	^∗∗∗^
37	ACV_sys_/ACA_dia_	0.023 (0.010)	0.047 (0.027)	41.9	56.0	^∗∗∗^
38	ACV_sys_/ACA_bl_	0.023 (0.010)	0.047 (0.026)	41.8	55.5	^∗∗∗^
39	ACV_dia_/ACA_dia_	0.018 (0.009)	0.040 (0.023)	50.2	58.4	^∗∗∗^
40	ACV_dia_/ACA_bl_	0.018 (0.009)	0.039 (0.023)	50.1	57.8	^∗∗∗^
41	RS_max_/RS	550.34E-5 (5.61E-5)	552.53E-5 (5.19E-5)	1.0	0.9	NS
42	RS/FS	–3.34 (0.70)	–2.88 (0.71)	21.0	24.5	^∗∗^
43	RS/ACA_bl_	4.94 (0.34)	4.72 (0.38)	6.9	8.0	^∗∗∗^
44	(A/ACA_bl_)/L_sys_	560.0 (87.5)	518.2 (83.8)	15.6	16.2	NS
45	(A/ACA_bl_)/L_dia_	170.7 (16.5)	182.0 (21.1)	9.7	11.6	^∗∗^
46	(A/ACA_bl_)/PPI_dia_	129.6 (8.8)	133.1 (10.1)	6.8	7.6	NS
47	(A_sys_/ACA_bl_)/L_sys_	151.8 (3.2)	155.9 (5.1)	2.1	3.3	^∗∗∗^
48	(A_sys_/ACA_bl_)/L_dia_	47.84 (10.61)	56.8 (13.0)	22.2	22.9	^∗∗∗^
49	(A_sys_/ACA_bl_)/PPI_dia_	36.02 (5.90)	41.14 (6.90)	16.4	16.8	^∗∗∗^
50	(A_dia_/ACA_bl_)/L_sys_	408.2 (86.3)	362.3 (81.8)	21.1	22.6	^∗^
51	(A_dia_/ACA_bl_)/L_dia_	122.9 (10.7)	125.2 (12.2)	8.7	9.7	NS
52	(A_dia_/ACA_bl_)/PPI_dia_	93.60 (8.80)	91.92 (8.87)	9.4	9.7	NS

*^∗∗∗^*P* < 0.001, ^∗∗^*P* < 0.01, ^∗^*P* < 0.05, NS, Not significant, Wilcoxon signed rank test with Bonferroni correction.*

**FIGURE 2:**
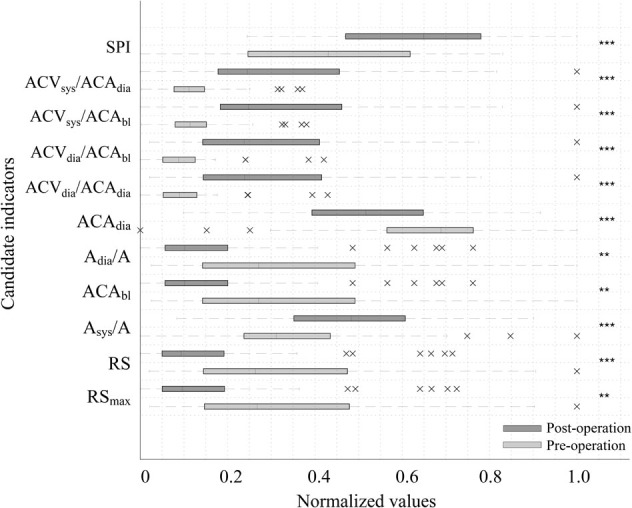
Boxplot of the top 10 accuracy parameters and SPI before and after surgery. The lighter box shows pre-operative results while the darker box shows post-operative results. Cross mark (“×”) represents the outlier. Crosses on the right side refer to outliers of more than 1.5 times the interquartile range above the third quartile. Crosses on the left-side refer to outliers of less than 3/2 times the size of the lower quartile. The spacings between different parts of the box indicate the degree of dispersion (spread) and skewness in the data. Every parameter shows a significant difference by surgical stress (^∗∗∗^*P* < 0.001, ^∗∗^*P* < 0.01).

**Figure 2** shows a boxplot of the top 10 parameters with high accuracy. The lighter box in the figure shows preoperative results, while the darker box shows post-operative results. The boxplot in **Figure 2** graphically depicts the groups of values of each parameter through their quartiles. Lines extending vertically from these boxes indicate variability outside the upper and lower quartiles. The cross mark (“×”) represents an outlier. Crosses on the right side refer to the outliers of more than 3/2 times the size of the upper quartile. Crosses on the left-side refer to the outliers of less than 3/2 times the size of the lower quartile. The spacing between different parts of the box indicates the degree of dispersion and skewness in the data.

### Classification Performance

Thirteen out of the 51 indices showed better performance than SPI in post-operative pain classification accuracy. Among these, the four indicators normalizing the difference of the adjacent pulse amplitudes, ACV_sys_/ACA_dia_, ACV_sys_/ACA_bl_, ACV_dia_/ACA_dia_, and ACV_dia_/ACA_bl_, showed remarkably high performance. The remaining top 10 accuracy of classification was higher in the order of pulse amplitude from the adjacent diastolic peak (ACA_dia_), normalized diastolic waveform area (A_dia_/A), pulse amplitude from baseline (ACA_bl_), normalized systolic waveform area (A_sys_/A), average slope of systolic interval (RS), and maximum value of systolic slope (RS_max_). **Table 5** shows the classification of the performance of parameters with statistically significant differences.

**Table 5:** Classification performance based on logistic classification.

Rank	Parameter	AC (%)	SE (%)	SP (%)	PPV (%)
1	ACV_sys_/ACA_dia_	79.50	74.02	85.99	84.54
2	ACV_sys_/ACA_bl_	78.25	71.82	85.71	83.95
3	ACV_dia_/ACA_dia_	76.41	66.21	87.20	83.99
4	ACV_dia_/ACA_bl_	76.28	66.90	86.39	83.52
5	ACA_dia_	66.75	78.16	56.04	64.37
6	A_dia_/A	66.75	66.17	69.53	69.13
7	ACA_bl_	66.69	77.26	56.35	65.70
8	A_sys_/A	66.56	64.87	69.61	69.16
9	RS	66.50	79.35	55.45	63.84
10	RS_max_	66.41	80.89	53.28	62.72
11	L_FS_	66.38	78.43	55.29	64.21
12	A_sys_	66.28	76.38	56.60	65.38
13	L_RS_	66.25	77.97	56.19	63.10
14	SPI	65.88	65.94	66.51	66.54
15	A_dia_	65.78	78.78	53.74	62.87
16	A_sys_/A_dia_	65.63	64.48	69.25	69.23
17	(A_sys_/ACA_bl_)/PPI_dia_	65.56	61.83	70.56	68.23
18	(A_sys_/ACA_bl_)/L_dia_	65.44	63.85	68.37	67.33
19	FS	65.31	77.37	54.37	63.91
20	TriA	65.22	77.17	54.59	62.82
21	TriA_sys_	65.22	76.30	55.83	61.79
22	A	64.94	77.85	53.06	62.53
23	TriA_dia_	63.84	77.85	51.08	60.80
24	TriA_sys_/TriA_dia_	63.75	60.69	68.25	65.88
25	TriA_dia_/TriA	63.47	60.94	68.34	66.78
26	A_sys_/ACA_bl_	63.31	58.07	69.90	66.39
27	L_sys_/L_dia_	63.13	59.53	68.07	64.51
28	L_dia_/PPI_dia_	63.00	61.99	65.02	64.41
29	L_sys_/PPI_dia_	62.66	61.76	65.78	65.06
30	(A_sys_/ACA_bl_)/L_sys_	62.63	61.97	64.54	62.53
31	RS/FS	62.47	67.04	59.65	62.65
32	L_sys_	62.38	58.54	67.10	63.63
33	TriA_sys_/TriA	62.25	62.54	63.46	62.24
34	RS/ACA_bl_	61.94	58.01	66.71	63.55
35	PW_30_	61.41	66.39	59.10	61.47
36	(A_dia_/ACA_bl_)/L_sys_	61.09	66.46	57.68	61.42
37	(A/ACA_bl_)/L_sys_	60.69	65.45	58.08	61.57
38	L_dia_	58.09	62.68	57.75	60.65
39	PW_90_	57.69	55.48	62.97	60.75
40	RS_max_/RS	57.38	55.64	63.42	61.91
41	(A/ACA_bl_)/L_dia_	56.22	53.10	61.49	58.08
42	PPI_dia_	56.16	60.90	55.66	58.70
43	PW_70_	55.88	54.08	60.34	58.72
44	PPI_sys_	55.34	61.21	54.53	58.00
45	A_dia_/ACA_bl_	55.28	60.59	53.71	56.56
46	(A_dia_/ACA_bl_)/PPI_dia_	51.75	53.40	55.92	53.35
47	A/ACA_bl_	48.53	53.36	50.39	51.58
48	(A/ACA_bl_)/PPI_dia_	47.22	50.54	48.47	48.28
49	ACV_dia_	46.91	41.59	58.11	48.92
50	(A_dia_/ACA_bl_)/L_dia_	45.25	45.66	53.40	50.88
51	PW_50_	42.38	47.01	46.60	37.03
52	ACV_sys_	41.94	49.60	44.32	34.17

## Discussion

### Possibility of PPG as a Post-operative Pain Indicator

Photoplethysmography is an optical technique used to determine the blood volume changes by measuring the amount of light that is transmitted, reflected, or scattered after the light has been irradiated to the peripheral site of the body. PPG is mainly measured at the fingertips, tips of toes, or earlobes. PPG has a larger or smaller value depending on the amount of blood ejected from the heart. It generally defines the upward convex pole as the systolic peak and the downward convex pole as the diastolic peak (Allen, 2007). Previous studies using PPG waveform analysis for surgical pain assessment have found that the PPG amplitude and systolic peak interval are significantly reduced according to the skin incision during propofol-remifentanil anesthesia (Rantanen et al., 2006). In another study it was found that the maximum slope between the diastolic pole and the systolic pole is significantly reduced according to the skin incision during fentanyl, propofol, and sevoflurane anesthesia (Seitsonen et al., 2005). Huiku et al. (2007) extracted normalized PPGA (PPGA_norm_), pulse transit time (PTT), systolic blood pressure (SBP), normalized heart beat interval (HBI_norm_), RE, and sympatho-vagal ratio (SVR) from PPG or other signals. After analyzing pain relevance with these parameters, they found that PPG_norm_, PTT, and SBP were highly correlated with pain stimuli (Huiku et al., 2007). Moreover, studies using state and RE, HR, and PPGA (Rantanen et al., 2006) or using HBI, SD1, SD2, SD1/SD2 of HRV Poincaré analysis results, RE, difference of RE and state entropy, PPGA, PPG notch amplitude, and PPG notch *y* position (Rantanen et al., 2006) have shown that SPI is superior to other indices.

Previous studies have revealed the relationship between PPG waveform characteristics and pain. However, further analytical methods are needed for pain assessment using PPG. The waveform of PPG can provide a range of hemodynamic information. For example, the perfusion index (the ratio of AC amplitude and DC level of PPG) is sensitive to proximal sympathectomy (Klodell et al., 2005), peripheral perfusion in critically ill patients (Lima et al., 2002), and neonatal left heart obstruction (Granelli and Östman-Smith, 2007). Stiffness index (defined as the amplitude ratio of the systolic peak and notch point) and reflection index (defined as systolic pole-to-notch position interval proposed by Millasseau et al. (2006) can be used to indirectly estimate the stiffness of the vessel. It has been reported that the ratio of the inflection points of the second derivative photoplethysmography (SDPTG) reflects the vascular stiffness due to aging (Takazawa et al., 1998). Thus, PPG morphology analysis reveals the possibility of extracting pain parameters with high significance, besides the parameters used in existing PPG-pain studies.

### Effectiveness of PPG-Based Parameters in Post-operative Pain Assessment

Similar to previous studies, A, A_sys_, and A_dia_ were significantly decreased after the operation (Seitsonen et al., 2005). Considering that the area-related parameter of the waveform reflects changes in blood volume at the measurement point, the reduction in area might be due to the contraction of blood vessels through sympathetic activation by pain. RS_max_, RS, L_RS_, FS, and L_FS_ associated with the up and down slopes of ACA_dia_, ACA_bl_, and PPG representing PPG amplitudes were also significantly reduced after the operation, which is consistent with the results of previous studies (Seitsonen et al., 2005; Rantanen et al., 2006; Kallio et al., 2008). These results can be explained by increased blood vessel resistance shown in pulse area change or decreased maximum blood volume due to vasoconstriction. From a similar viewpoint, significant increase in L_sys_ indicates that contracted vessels might have increased the vascular resistance due to vasoconstriction of the sympathetic nerves (Jaryal et al., 2009; Hickey et al., 2016). TriA, TriA_sys_, and TriA_dia_ in the area of a triangle where the waveform of the high frequency component such as harmonics was removed showed similar patterns as those of A, A_sys_, and A_dia_, respectively. This might indicate the lack of significant correlation between pain and the high frequency component of PPG. HBI derived from systolic and diastolic extremes known to be pain-related was increased after the operation but in our study the increase was not statistically significant. While this result is not common, the previous research also showed that HBI has relatively low sensitivity and specificity compared with combined feature of PPG (Seitsonen et al., 2005; Rantanen et al., 2006).

In the case of PW, PW_30_, PW_50_, and PW_70_ showed no significant change between pre- and post-operation. However, PW_90_ was significantly increased in post-operation. Changes in PW_90_ might be due to the narrowing of the waveform at the upper part of the amplitude when pain occurred. This can be interpreted in terms of increasing systolic interval length. Physiologically, the increase of systolic length increased the severity of vascular flow resistance (Angius et al., 2012; Wang et al., 2015).

### Normalization

The normalization in this study is divided into two types: that for the spatial-domain of PPG such as amplitude and that for the temporal domain of the PPG waveform such as interval or length. First, on the spatial domain, since the amplitude has an arbitrary value as mentioned above, it has an inter-individual difference as well as an intra-individual difference for each measurement. Thus, we derived ratio-based indices that could compensate for the arbitrary change of the amplitude. Secondly, on the temporal dimension, since the basal heart has individual difference, it is impossible to determine whether or not the pain is based on a specific HR. Moreover, every temporal value such as the systolic and diastolic lengths also depends on the beat-to-beat interval. However, the percentage of systolic interval during HR may be uniform for every person compared with the HR.

As a result of normalization, we confirmed that CVs were decreased in most of the indices. In the spatial domain, CV of A_sys_ and A_dia_ was decreased by dividing with A from 62.1 to 15.8% and from 70.0 to 6.1%, respectively. In the temporal domain, the CV of L_dia_ was decreased from 20.1 to 5.2%. Interestingly, the CV of the L_sys_ was increased by normalization (7.5–16.8%). This result could suggest that systolic length is less effected by the pulse length, and this concurs with the result of previous research that investigates systolic length change according to the exercises (Franculescu et al., 1982). In ACV_sys_ and ACV_dia_, CVs were decreased by normalization from 65.9 to 41.9% and from 67.5 to 41.8%, respectively. These results suggest that the normalization could reduce the intra-individual and inter-individual differences. However, for a more generalized result, normalization with a massive dataset is needed.

### Dynamic Parameters

The results of pain classification using these proposed parameters showed that parameters derived from pulse-to-pulse analysis and normalized parameters were superior to those derived from single pulse analysis. For example, the classification accuracy of parameters such as ACV_sys_/ACA_bl_, ACV_sys_/ACA_dia_, ACV_dia_/ACA_bl_, and ACV_dia_/ACA_dia_ reflecting the dynamic changes between pulses was about 80%, which was more than 10% higher than the accuracy when single parameters were used without normalization. These dynamic parameters can resemble blood pressure variability (BPV). BPV is known to reflect blood pressure variations in very short-term analysis (beat-to-beat), which influences the central and reflex autonomic modulation in short term analysis (<24 h) (Conway et al., 1984; Mancia et al., 1986; Parati et al., 1995) and the elastic properties of arteries (reduced arterial compliance) (Parati, 2005; Kotsis et al., 2011; Fukui et al., 2013). However, the relationship between BPV and pain has not yet been reported. Therefore, it is more reasonable to conclude that dynamic parameters of amplitude variation can distinguish the pain better than static parameters, since stroke volume variability (SVV), a dynamic parameter, can reflect volume responses more effectively than the static parameters HR and BP in hemodynamic analysis.

### Collinearity Between Features

**FIGURE 3:**
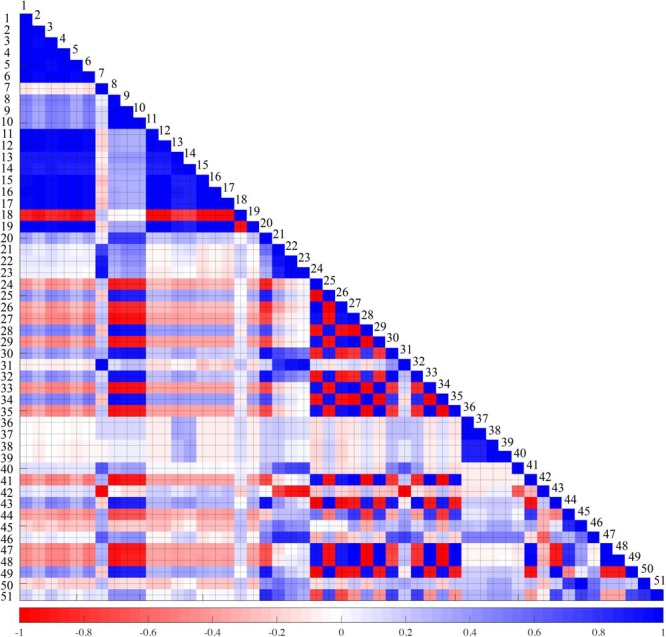
Collinearity test result between 51 features. Blue and red represents positive and negative correlation, respectively. The darker the color, the higher the correlation. 1: A, 2: A_sys_, 3: A_dia_, 4: TriA, 5: TriA_sys_, 6: TriA_dia_, 7: L_sys_, 8: L_dia_, 9: PPI_sys_, 10: PPI_dia_, 11: ACA_dia_, 12: ACA_bl_, 13: ACV_sys_, 14: ACV_dia_, 15: RS, 16: L_RS_, 17: RS_max_, 18: FS, 19: L_FS_, 20: PW_30_, 21: PW_50_, 22: PW_70_, 23: PW_90_, 24: A_sys_/A, 25: A_dia_/A, 26: A_sys_/A_dia_, 27: TriA_sys_/TriA, 28: TriA_dia_/TriA, 29: TriA_sys_/TriA_dia_, 30: A/ACA_bl_, 31: A_sys_/ACA_bl_, 32: A_dia_/ACA_bl_, 33: L_sys_/PPI_dia_, 34: L_dia_/PPI_dia_, 35: L_sys_/L_dia_, 36: ACV_sys_/ACA_dia_, 37: ACV_sys_/ACA_bl_, 38: ACV_dia_/ACA_dia_, 39: ACV_dia_/ACA_bl_, 40: RS_max_/RS, 41: RS/FS, 42: RS/ACA_bl_, 43: (A/ACA_bl_)/L_sys_, 44: (A/ACA_bl_)/L_dia_, 45: (A/ACA_bl_)/PPI_dia_, 46: (A_sys_/ACA_bl_)/L_sys_, 47: (A_sys_/ACA_bl_)/L_dia_, 48: (A_sys_/ACA_bl_)/PPI_dia_, 49: (A_dia_/ACA_bl_)/L_sys_, 50: (A_dia_/ACA_bl_)/L_dia_, 51: (A_dia_/ACA_bl_)/PPI_dia_

The aim of this study is to analyze the many features of PPG, and to identify the indicators that correlate with pain. Thus, a total of 51 indicators were derived, which include interdependent indicators. Indicator collinearity does not need to be treated as important when single indicators are used to classify pain. However, as in the case of the SPI, if collinearity is used to generate a combined indicator with some indicators, it may be necessary to evaluate the collinearity between the two indicators, since this may lead to errors in accuracy evaluation. The index collinearity is usually assessed using the condition number, variance inflation factor (VIF) or correlation coefficient. In this study, the collinearity between each index was calculated from the correlation coefficient, and is shown in **Figure 3**. In the figure, blue is the positive correlation coefficient and red is the negative correlation coefficient. The color concentration reflects the magnitude of the correlation coefficient. The results of this collinearity evaluation can be used in developing combined indicators using single indicators.

### Limitations

The aim of our study is to evaluate individual predictors rather than regression by combining multiple predictors. If we develop a combination feature that combines individual features in the future, we should perform a multicollinearity test between the features found in this study. Also, in order for the study to be a useful index in clinical practice, it is necessary to derive a pain index discriminant formula that can express the pain score as a normalized value. Finally, this study does not include an assessment of the pain situation suppressed by analgesics. Therefore, it is necessary to verify whether the post-operative pain candidate index reflects real-time pain change following analgesic administration in order to be used clinically in post-operative pain evaluation. Moreover, a composite test for a specific age, disease, etc., is required to improve reliability.

## Conclusion

Results of this study demonstrated that dynamic parameters obtained by inter-pulse analysis were more sensitive to pain than static parameters obtained from single pulse analysis. Considering that existing surgical pain assessment studies are performed mainly on the above mentioned static indicators, dynamic indicators found in this study can be used to develop more sophisticated pain assessment algorithms in the future. However, further studies are needed before these new pain parameters identified in this study can be applied to clinical practice, including evaluation in general anesthesia. First, this study was based on a statistical analysis of total signals for 6 min obtained before and after surgery without observing changes in pain in real-time. Therefore, it is necessary to determine whether real-time pain change estimation is possible. In addition, this study only dealt with single parameters obtained from PPG waveforms. Further studies with multivariate models using multiple indicators are needed for more sophisticated pain assessment. Finally, since the pain assessment technique must be provided as a formula that can quantify the degree of pain, further research is needed to determine the normalization technique to correct inter-individual differences.

## Author Contributions

B-MC and G-JN collected the data. HS designed the data analysis scheme. YLY, HSS, and HS performed the data analysis. B-MC and HS interpreted the results. All authors contributed to the writing of the manuscript, provided critical revisions, and approved the final version.

## Conflict of Interest Statement

The authors declare that the research was conducted in the absence of any commercial or financial relationships that could be construed as a potential conflict of interest.

## Footnotes

1 http://cris.nih.go.kr
